# Machiavellianism and Intimate Partner Violence Perpetration: A Systematic Review and Meta-Analysis

**DOI:** 10.1177/15248380241270027

**Published:** 2024-08-20

**Authors:** Lisa K. White, Natasha Valos, Xochitl de la Piedad Garcia, Megan L. Willis

**Affiliations:** 1 School of Behavioural and Health Sciences, Australian Catholic University, Strathfield, NSW, Australia

**Keywords:** Machiavellianism, Dark Triad, intimate partner violence, domestic violence, meta-analysis

## Abstract

This systematic review and meta-analysis estimated the size of the relationship between Machiavellianism and intimate partner violence (IPV) perpetration. Further, we explored whether the strength of this relationship varied depending on IPV type (i.e., physical, psychological, sexual, and cyber), and perpetrator gender. Systematic searches of Medline Complete, PsycInfo, Scopus, and Web of Science databases were conducted on July 4, 2023. Studies were included if they were a peer-reviewed published paper or unpublished dissertation, in English, included a measure of Machiavellianism and IPV perpetration, and reported the relationship between these variables. Study quality was assessed using the AXIS tool. Nineteen studies (*N* = 9,464) were included in a random-effects meta-analysis revealing a significant, weak, positive correlation between Machiavellianism and IPV perpetration (*r* = .16, 95% CI [0.11, 0.21], *p* < .001). Machiavellianism had a significant, weak, positive relationship with cyber (*r* = .25, 95% CI [0.17, 0.32], *p* < .001), psychological (*r* = .20, 95% CI [0.15, 0.24], *p* < .001), and sexual IPV (*r* = .10, 95% CI [0.02, 0.19], *p* = .020). No significant relationship was found for physical IPV. There was no significant difference in the strength of the relationship between women and men. These findings are limited by the measures being self-report, heterogeneity across studies, and the cross-sectional nature of the included studies which limits the understanding of causal pathways. Nonetheless, this meta-analysis shows a link between Machiavellianism and IPV perpetration, and future research should examine how this knowledge may be used to reduce IPV perpetration.

The World Health Organization defines intimate partner violence (IPV) as any behavior within an intimate relationship that results in physical, sexual, or psychological harm. Such behaviors include but are not limited to, physical or sexual violence; emotional abuse including threats of harm, belittling, or intimidation; and acts intended to control the other person such as restricting access to friends and resources or monitoring movements (World Health Organisation, 2012). These acts may be perpetrated either in person or through technology in what is referred to as cyber IPV (Gilbar et al., 2023). Such technologically based IPV may include behaviors such as using phone applications to track a person, nonconsensually disseminating private photographs, or using online platforms to harass an intimate partner (Pineda et al., 2022).

## Prevalence and Impacts of IPV

IPV is a serious public health concern given the alarming prevalence and the significant impacts it has on those exposed to it. Global estimates indicate that at least 4 in 10 women (White et al., 2024), and one in five men (Desmarais et al., 2012) will be subjected to IPV within their lifetime. Although it should be noted that actual prevalence rates may differ because of under-reporting due to factors such as cultural norms, feelings of shame, and a lack of support services (White et al., 2024). The effects of IPV are wide ranging, including risk of serious physical injury, employment instability, homelessness, and in some instances, death (Crowne et al., 2011; Stöckl et al., 2013; Wu et al., 2010; Yakubovich et al., 2022). From a psychological standpoint, exposure to IPV is associated with many adverse outcomes including depression, anxiety, post-traumatic stress disorder, substance use, and low self-esteem (Karakurt et al., 2014; Spencer et al., 2019). Beyond these individual-level outcomes, IPV has implications on the broader community and economy due to its impact on resources such as the health system, publicly funded housing, and emergency services (Baker et al., 2009; Peterson et al., 2018).

Accordingly, there is a clear need to better understand the risk factors of IPV perpetration. Such research may contribute to the development of targeted psychological interventions to support individuals who are exposed to IPV, while also informing how clinicians assess and work with perpetrators to reduce recidivism and improve outcomes.

## The Dark Triad of Personality: Machiavellianism

Personality research is one avenue that has gained popularity in the field of IPV to better understand the profile of those who perpetrate it. Of note, several researchers have focused on the Dark Triad of personality (Kanemasa et al., 2023; Kiire, 2017; March et al., 2020). The Dark Triad is comprised of the malevolent traits of narcissism, psychopathy, and Machiavellianism (Christie & Geis, 1970). These traits overlap yet are theoretically distinct: narcissism consists of inflated self-importance, entitlement, and grandiosity; psychopathy relates to a lack of empathy and impulsivity; and Machiavellianism is characterized by manipulation, coldness, exploitation of others for personal gain, and a disregard for morality (Christie & Geis, 1970; Millis-Koonce et al., 2023).

More recently, Machiavellianism has been researched as a bidimensional trait, consisting of the dimensions of Machiavellian views and Machiavellian tactics (Monaghan et al., 2020). The Machiavellian views dimension reflects a cynical, untrusting outlook of the world, while the Machiavellian tactics dimension captures an end justifies the means mentality. Factor analytic research has shown the bidimensional model to be a more psychometrically robust solution when investigating Machiavellianism (Monaghan et al., 2016). Further, such research has shown differences in how each dimension relates to other psychological constructs, with Machiavellian views being primarily associated with low self-esteem, emotional instability, and misanthropy, whereas Machiavellian tactics are correlated with interpersonal exploitation, low conscientiousness, and low reciprocity (Monaghan et al., 2020).

Unsurprisingly, the Dark Triad has been linked to a range of antisocial behaviors (Chen, 2010; Maloney et al., 2023). Of note, recent meta-analyses have estimated the relationship between two of the Dark Triad traits and IPV. First, Robertson et al. (2020) found a significant, positive, small correlation between psychopathy and IPV perpetration, both across effect sizes controlling (*r* = .15, 95% CI [0.09, 0.30], *k* = 10, *N = 2,786*) and not controlling (*r* = .20, 95% CI [0.07, 0.23], *k* = 14, *N* = 4,600) for covariates. Second, Oliver et al. (2023) also found a significant, small, positive correlation between narcissism and IPV perpetration (*r* = .15, 95% CI [0.12, 0.19], *k* = 33, *N* = 11,520). However, to date, no review has examined the relationship between Machiavellianism and IPV perpetration and there are no published meta-analytic estimates of the effect size of this relationship. This is a shortfall in the literature given research has shown that, unlike the other two traits, Machiavellianism is markedly influenced by environmental factors and thus to some extent, may be a trait that is learned (Jones & Mueller, 2022; Vernon et al., 2008). As such, it can be argued that Machiavellianism may be the most modifiable of the three traits (Furnham et al., 2013), and therefore, the most relevant to clinical practice, particularly in relation to how clinicians may psychologically formulate and implement interventions to effectively target the expression of this trait and the associated antisocial behavior.

## Machiavellianism and IPV Perpetration

Numerous studies have examined Machiavellianism in the context of IPV, however there have been inconsistent findings. For example, there have been several studies that have shown evidence in support of a significant, weak, positive correlation between Machiavellianism and IPV perpetration (Brewer & Abell, 2017; Kanemasa et al., 2022; March et al., 2020; Mayshak et al., 2020). However, other studies have reported conflicting findings, failing to show an association between the two variables (Bhogal & Wallace, 2022; Brewer & Abell, 2015; Plouffe et al., 2022a). Accordingly, there is a need for further research to clarify these mixed outcomes and to confirm whether a relationship exists between Machiavellianism and IPV perpetration.

One potential explanation for these inconclusive findings may be that Machiavellianism is differentially related to the various forms of IPV. Indeed, studies have examined either cyber, psychological, sexual, or physical IPV, or a combination of these IPV types, and have found different results across these manifestations of IPV. In particular, several studies have demonstrated a significant relationship between Machiavellianism and nonphysical types of IPV including cyber IPV (Smoker & March, 2017) and psychological IPV (Kanemasa et al., 2023). In contrast, others have failed to reveal a significant association with Machiavellianism when examining physical IPV (Carter & Egan, 2022; Kiire, 2017; Plouffe et al., 2022a). This suggests that the strength of the relationship between Machiavellianism and IPV perpetration may be moderated by IPV type.

Finally, research has shown mixed gender findings when examining the relationship between Machiavellianism and IPV perpetration. Some studies have shown that the relationship between Machiavellianism and IPV perpetration is significant among women, but not men (Kiire, 2017), whereas others have found this relationship to be stronger for men, than women (March et al., 2022). Thus, there is a need to further explore whether there is a relationship between Machiavellianism and IPV perpetration for both men and women and to determine whether there are differences in the magnitude of this relationship.

## Objectives

The primary aim of this systematic review and meta-analysis was to estimate the size of the relationship between Machiavellianism and IPV perpetration. It was hypothesized that a significant, positive correlation would be found between IPV perpetration and Machiavellianism. The secondary aim was to identify if any studies used a bifactor model of Machiavellianism and if there were sufficient studies, to determine whether the strength of the relationship differed between the two Machiavellianism dimensions: views and tactics. As highlighted, given the tactics dimension is more concerned with externalizing behaviors comparative to the views dimension, it was hypothesized that Machiavellian tactics would have a stronger relationship with IPV perpetration compared to Machiavellian views. The third aim was to examine whether the relationship between Machiavellianism and IPV perpetration varied depending on the IPV type (i.e., physical, psychological, sexual, and cyber). It was hypothesized that there would be differences in the strength of the relationship between Machiavellianism and the type of IPV perpetrated. Lastly, given the inconsistent findings in relation to gender, the final aim was to investigate whether the strength of the relationship between Machiavellianism and IPV perpetration differed for women and men.

## Method

### Search Strategy

This review was conducted in accordance with the Preferred Reporting Items for Systematic Review and Meta-Analysis guidelines (Page et al., 2021) and was registered with PROSPERO (CRD42023442532). Searches were conducted on July 4, 2023 using Medline Complete, PsycInfo, Scopus, and Web of Science databases with no date restrictions imposed. Terms for the concepts of Machiavellianism and IPV perpetration were searched for in the title and abstract fields. The following search terms were used for (a) Machiavellianism (“Machiavellian*” OR “dark triad” OR “dark tetrad” OR “dark personalit*”) and (b) IPV perpetration (“intimate partner*” OR “interpersonal violence” OR “relationship quality” OR “spous* violence” OR “spous* abuse” OR “spous* assault” OR “batter*” OR “marital violence” OR “marital abuse” OR “marital assault” OR “domestic violence” OR “domestic abuse” OR “domestic assault” OR “family violence” OR “family abuse” OR “family assault” OR “dating violence” OR “dating abuse” OR “dating assault” OR “psychological violence” OR “psychological abuse” OR “emotional violence” OR “emotional abuse” OR coerci* OR gaslight* OR “sexual violence” OR “sexual abuse” OR “sexual assault” OR “physical violence” OR “physical assault” OR “physical abuse” OR “cyber*”). The complete search strategy is provided in Supplemental Material. Manual searching of the article reference lists was also conducted to identify additional papers.

### Selection Process

Searches were imported to the Covidence review platform and automatically deduplicated. Title and abstract screening were conducted by LW and NV, followed by full-text screening. All discrepancies were resolved through discussion with MW.

### Eligibility Criteria

Studies were included if they (a) were available in English; (b) were published in a peer-reviewed journal or an unpublished dissertation; (c) used a validated Machiavellianism measure; (c) used any measure of IPV perpetration (e.g., validated scale, court records, and police reports); and (d) reported a statistical effect size estimate of the relationship between Machiavellianism and IPV perpetration. Studies were excluded if they (a) were a book chapter, review, qualitative paper, or conference abstract; (b) were not specific to violence perpetrated against an intimate partner (e.g., sexual assault perpetrated against a stranger); (c) reported the relationship between IPV and a global measure of dark personality, rather than with Machiavellianism alone; or (d) were an unpublished duplicate of an included published paper.

### Data Extraction

Data was extracted by LW and reviewed by MW. Data extracted into a Microsoft Excel spreadsheet included (a) author(s); (b) publication year; (c) study location; (d) sample size; (e) population type; (f) age; (g) gender; (h) ethnicity; (i) education; (j) Machiavellianism measure; (k) IPV perpetration type(s); (l) IPV perpetration operationalization; and (m) correlation coefficients of the relationship between Machiavellianism and IPV perpetration. Only the baseline data of longitudinal studies was extracted for inclusion. One author, Gamache et al. (2022), was emailed to obtain data for the variables of interest. Gamache et al. (2022) provided the relevant data which was collected from a subsample of their study.

### Quality Assessment and Risk of Bias

As most studies were cross-sectional, the AXIS quality appraisal tool (Downes et al., 2016) was used to assess quality. LW and NV assessed each study with discrepancies resolved through discussion. The AXIS tool (Downes, et al., 2016) assesses five key domains including the introduction (objectives), methods (sample size, population, etc.), results (internal consistency, nonresponse bias, etc.), discussion (conclusions justified, limitations outlined, etc.) and ethical issues (conflicts of interest and informed consent). It comprises 20 questions requiring a response of “yes”, “no”, or “don’t know”. Items marked “no” or “don’t know” received a score of 0 and items marked “yes” received a score of 1, except for items 13 and 19 which were reverse scored (items awarded one point were converted to zero and vice versa) to meet the methodological intent. Items marked as “not applicable” were awarded one point to ensure papers were not penalized for irrelevant items. Downes et al. (2016) do not provide guidance on scoring, thus we followed Oliver et al.’s (2023) approach, where total scores were computed and categorized as low (0–7), medium (8–14), or high quality (15–20).

### Data Analyses

A random-effects meta-analysis of correlation coefficient effect sizes was conducted using the Comprehensive Meta-Analysis (CMA) Version 4 software (Borenstein et al., 2022). As per Cohen (1998), effect sizes were interpreted as weak (*r* = .10–.29), moderate (*r* = .30–.49), or strong (*r* = .50 or greater). Heterogeneity was examined using the *Q* and *I*^2^ statistics. Higgins et al. (2003) suggest that *I*^2^ statistics above 25% are indicative of low heterogeneity, above 50% indicates medium heterogeneity and above 75% indicates high heterogeneity. Studies were considered heterogeneous if the *Q* statistic was significant (*p* < .05). Publication bias was assessed with funnel plots and Egger’s test using a *p*-value of less than .05 (Egger et al., 1997). Duval and Tweedie’s (2000) trim-and-fill analysis and the classic failsafe *N* test were also used.

#### Overall Relationship

The main analysis examined the overall effect size between Machiavellianism and IPV perpetration. When a study reported effect sizes for the relationships between Machiavellianism and multiple IPV types, CMA was used to estimate the average effect before entering this value into the meta-analysis. When studies reported data for men, women, and a gender-combined sample, only the combined effect size was included. When studies did not report a gender-combined effect, CMA was used to average the effect size between the men and women to produce an overall effect.

#### Subgroup Analyses

Three subgroup analyses were planned, following the aims of our study. The first subgroup analysis aimed to determine whether the strength of the relationship between Machiavellianism and IPV perpetration differed between the two subtypes (views and tactics). The second subgroup analysis examined the relationships between Machiavellianism and each IPV type, for which there were at least three effect size estimates. When IPV type was unspecified, it was coded as multidomain IPV and not included in this subgroup analysis. Studies that included effect sizes for multiple outcome measures for the same IPV type were averaged using CMA to produce an overall effect. The third subgroup analysis explored gender differences in the relationship between Machiavellianism and IPV perpetration.^1^ Studies that only captured data from one gender were included in this analysis.

## Results

**Figure 1.**
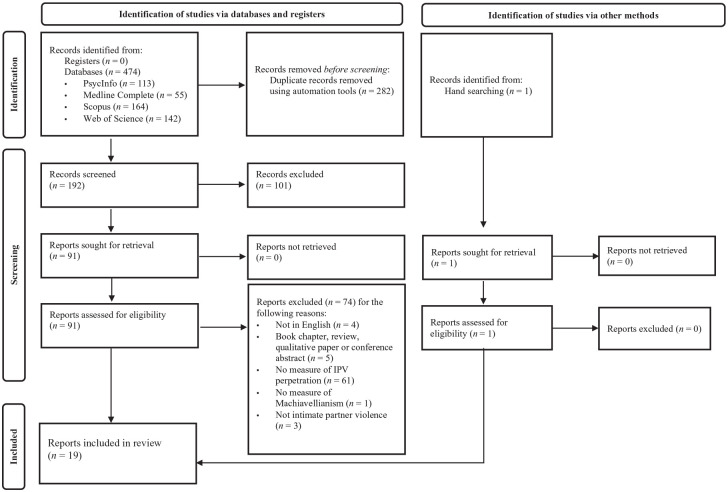
Preferred Reporting Items for Systematic Reviews and Meta-Analyses (PRISMA) flow diagram of the study selection process.

Figure 1 outlines the search and screening process. A total of 474 articles were identified through database searches. One additional article was found through hand searching. After deduplication and screening, a total of 18 articles met the inclusion criteria. One article contained two studies, resulting in a final sample of 19 studies for inclusion.

### Sample and Study Characteristics

Table 1 summarizes the sample characteristics of the included studies. All studies were completed between 2015 and 2023 with participants recruited from the United Kingdom (*k* = 5), Canada (*k* = 2), Japan (*k* = 4), Australia (*k* = 3), the United States of America (*k* = 1), and Spain (*k* = 1). Others recruited samples from a combination of countries including the United States of America and Canada (*k* = 2), and the United Kingdom and Sweden (*k* = 1). A total of 9,464 participants were recruited from community samples (*k* = 13), university samples (*k* = 4), or a combination of both (*k* = 2). Most studies recruited predominantly women (*k* = 12) while the remaining studies consisted of a relatively even gender split (*k* = 7). Several studies did not report the ethnicity of participants (*k* = 9) and of those that did, the majority used samples that were largely comprised of white participants (*k* = 7). Likewise, most studies (*k* = 10) did not report the education level of participants. Studies that did include this information had recruited samples where most of the participants had either commenced or completed tertiary education (*k* = 9). Most studies recruited samples that were predominantly heterosexual (*k* = 11). The other eight studies did not report information about participant sexuality. The mean age across all studies ranged from 18.74 to 44.97 years.

**Table 1.** Sample and Study Characteristics of the Included Studies.

Author(s) (year), Country	Sample type, *n*	% Men	Age, *M* (*SD*)	% White	% Tertiary educated	% Heterosexual	IPV type(s)						IPV measure(s)	Mach measure
							Multidomain^a^	Physical	Psychological	Sexual	Cyber	Other		
Bhogal and Wallace (2022), UK	C/U, 124	30.4	30.7 (9.2)	NR	NR	100					✓		CDAQ	SD3
Brewer and Abell (2015), UK	C, 234	39.7	26.2 (9.0)	68.40	NR	100				✓			SCIRS	MACH-IV
Brewer and Abell (2017), UK	C, 132	0.0	25.7 (8.6)	NR	NR	100			✓				MMEA	MACH-IV
Carter and Egan (2022), UK	C, 416	23.1^b^	28.0 (9.5)	45.0	NR	70.9		✓	✓				CTS-2; MMEA	SD4
Carton and Egan (2017), UK	C, 128	18.0^b^	NR	89.8	NR	93.0	✓		✓				CTS-2; MMEA	SD3
Gamache et al. (2022), Canada	C/U, 377	16.4^c^	24.4 (3.4)^c^	NR	90.4	NR		✓	✓	✓			CTS-2	DTDD
Kanemasa et al. (2022), Japan	C, 1392	43.7	29.7 (5.9)	NR	73.5	NR			✓				IPIPV	DTDD
Kanemasa et al. (2023), Japan	C, 942	50.0	45.0 (8.9)^d^	NR	NR	100			✓				IPIPV	DTDD
Kiire (2017), Japan	U, 344	47.10^b^	19.0(1.3)	0.00	100.0	NR	✓	✓	✓	✓		✓	IPVS	SD3
Kiire (2019), Japan	U, 380	44.2	18.9 (1.2)	NR	100.0	NR		✓	✓	✓		✓	IPVS	SD3
March et al. (2020), Australia	C, 405	30.4	24.7 (7.3)	84.00	NR	73.8			✓		✓		IPCS; CBS-R	MACH-IV
Mayshak et al. (2020), Australia	C, 1009	45.9^b^	32.3 (13.4)	NR	>50.0^e^	NR	✓						Other survey^f^	SD3
Millis-Koonce et al. (2023), USA	C, 203	0.00	30.8 (5.2)	NR	NR	100		✓	✓				CTS	SD3
Pineda et al. (2022), Spain	C, 1189	21.95	29.4 (10.5)	NR	61.8	NR					✓		CDAQ	SD3
Plouffe et al. (2022a), USA/Canada	C, 399	38.35	33.5 (10.3)	77.9	90.3	83.5		✓					Other survey^g^	MACH-IV
Plouffe et al. (2022b; Study 1), Canada	U, 399	27.32	18.7 (1.8)	55.9	100	100		✓	✓				CTS-2	MACH-IV
Plouffe et al. (2022b; Study 2), USA/Canada	C, 360	42.50	34.4 (11.0)	80.6	88.6	100		✓					Other survey^g^	MACH-IV
Smoker and March (2017), Australia	C, 689	30.00	26.0 (10.2)	NR	NR	NR					✓		IPCS	SD3
Tetreault et al. (2021), UK/Sweden	U, 342	40.35	26.5 (7.7)	90.6	NR	NR		✓	✓				CTS	SD3

*Note.* C = Community sample; U = University sample; NR = not reported; CDAQ = Cyber Dating Abuse Questionnaire (Borrajo et al., 2015); SCIRS = Sexual Coercion in Intimate Relationships Scale (Shackelford & Goetz, 2004); MMEA = Multidimensional Measure of Emotional Abuse (Murphy & Hoover, 1999); CTS-2 = The Revised Control Tactics Scales - Short Form (Straus & Douglas, 2004); IPIPV = Indirect and Psychological Intimate Partner Violence Scale (Soma et al., 2004); IPVS = Intimate Partner Violence Scale (Kiire, 2017); IPCS = Intimate Partner Cyberstalking Scale (Smoker & March, 2017); CBS-R = Revised Controlling Behaviors Scale (Graham-Kevan & Archer, 2005); CTS = Conflict Tactics Scales (Straus, 1979); SD3 = Short Dark Triad (Jones & Paulhus, 2014); MACH-IV = Machiavellianism Scale (Christie & Geis, 1970); SD4 = Short Dark Tetrad (Paulhus et al., 2021); DTDD = Dark Triad Dirty Dozen scale (Jonason & Webster, 2010); IPV = intimate partner violence.

a Coded as multidomain IPV when studies combined a variety of IPV types.

b Respective studies reported sex rather than gender.

c Reported data is from larger sample.

d Reported data is an average of men and women in study.

e Exact percentage was not reported.

f Survey adapted from the Australian Bureau of Statistics (ABS) Personal Safety Survey (ABS, 2016).

g Survey adapted from evaluation procedure implemented by Graham et al. (2012).

Table 1 also summarizes the study characteristics of the included studies. Nearly all used a cross-sectional design except for two longitudinal studies (Kanemasa et al., 2022, 2023). Several measures were used to operationalize Machiavellianism. The most frequently used was the Short Dark Triad (Jones & Paulhus, 2014, *k* = 9), followed by the Machiavellianism Scale (Christie & Geis, 1970, *k* = 6), and the Dark Triad Dirty Dozen scale (Jonason & Webster, 2010, *k* = 3). One study used the Short Dark Tetrad (Paulhus et al., 2021). Several studies operationalized IPV as a multidomain construct by measuring multiple IPV types (*k* = 3). Others examined specific IPV types, with several studies assessing more than one type (*k* = 9). The most frequently investigated was psychological (*k* = 12), followed by physical (*k* = 9), sexual (*k* = 4) and cyber (*k* = 4).

A range of scales were used to measure IPV perpetration. The most frequently used was the Revised Control Tactics Scales—Short Form (Straus & Douglas, 2004, *k* = 5), followed by the Multidimensional Measure of Emotional Abuse (Murphy & Hoover, 1999, *k* = 3), the Cyber Dating Abuse Questionnaire (Borrajo et al., 2015, *k* = 2), the Intimate Partner Violence Scale (Kiire, 2017, *k* = 2), and the Intimate Partner Cyberstalking Scale (Smoker & March, 2017, *k* = 2). The Indirect and Psychological Intimate Partner Violence Scale (Soma et al., 2004) was used by Kanemasa et al. (2022, 2023). Kanemasa et al. (2022) collected data from the perpetrator, whereas Kanemasa et al. (2023) used partner reports of IPV. Three studies used other surveys; two used an adapted questionnaire implemented by Graham et al. (2012) which included several items about the use of physical violence, and the other used a questionnaire adapted from the ABS Personal Safety Survey (ABS, 2016). All other IPV measures were used only once.

### Quality Assessment and Risk of Bias

Table 2 summarizes the quality assessment ratings. The average rating across studies was *M* = 17.58, *SD* = 2.21, indicating that overall, the studies were of high quality. The majority of quality issues were associated with items 3, 5, 7, and 19. Of the 19 studies, 12 (63%) did not conduct a power analysis to determine a minimum sample size. As such, there is a risk that these studies were underpowered and thus vulnerable to a type II error, where random error may have led to an undetected true effect. Seven studies (37%) recruited samples that were restricted by unique characteristics, such as recruiting university students or women who were pregnant, thereby limiting generalisability. Three studies (16%) did not report or describe missing data. In these studies, it was unclear whether there was a systematic pattern to the omitted items, which increased the risk that the findings were compromised by nonresponse bias. Finally, six studies (32%) failed to report funding sources, and it was unclear whether conflicts of interest may have affected the interpretation of results. Despite these limitations, the overall study quality ranged from medium to high quality and thus all studies were included in the meta-analysis.

**Table 2.** Quality Assessment and Risk of Bias (Inter-rater Reliability for Assessment was 98.85%).

Author(s) (year)	Intro	Method										Results					Discuss		Ethics		Rating
	Question number																				
	1	2	3	4	5	6	7	8	9	10	11	12	13*	14	15	16	17	18	19*	20	
Bhogal and Wallace (2022)	1	1	0	1	1	1	N/A	1	1	1	1	1	N/A	N/A	1	1	1	1	1	1	19
Brewer and Abell (2015)	1	1	0	1	1	1	N/A	1	1	1	1	1	N/A	N/A	1	1	1	1	DK	1	18
Brewer and Abell (2017)	1	1	0	1	1	1	N/A	1	1	1	1	1	N/A	N/A	1	1	1	1	1	DK	18
Carter and Egan (2022)	1	1	1	1	1	1	1	1	1	1	1	1	1	0	1	1	1	1	1	1	19
Carton and Egan (2017)	1	1	1	1	1	1	N/A	1	1	1	1	1	N/A	N/A	1	1	1	1	DK	1	19
Gamache et al. (2022)	1	1	0	1	1	0	0	1	1	1	1	0	0	0	1	0	1	1	1	1	13
Kanemasa et al. (2022)	1	1	1	1	1	1	N/A	1	1	1	1	1	N/A	N/A	1	1	1	1	1	1	20
Kanemasa et al. (2023)	1	1	1	1	1	1	N/A	1	1	1	1	1	N/A	N/A	1	1	1	1	1	1	20
Kiire (2017)	1	1	0	1	0	0	N/A	1	1	1	1	1	N/A	N/A	1	1	1	1	DK	1	16
Kiire (2019)	1	1	0	1	0	0	0	1	1	1	1	1	0	0	1	1	1	1	DK	1	13
March et al. (2020)	1	1	1	1	1	1	N/A	1	1	1	1	1	N/A	N/A	1	1	1	1	DK	1	19
Mayshak et al. (2020)	1	1	1	1	0	0	1	1	1	1	1	1	1	1	1	1	1	1	1	1	18
Millis-Koonce et al. (2023)	1	1	1	1	0	0	N/A	1	1	1	1	1	N/A	N/A	1	1	1	1	1	1	18
Pineda et al. (2022)	1	1	0	1	1	1	N/A	1	1	1	1	1	N/A	N/A	1	1	1	1	1	1	19
Plouffe et al. (2022a)	1	1	0	1	0	0	1	1	1	1	1	1	1	N/A	1	1	1	1	1	1	17
Plouffe et al. (2022b; Study 1)	1	1	0	1	1	1	1	1	1	1	1	1	1	N/A	1	1	1	1	1	1	19
Plouffe et al. (2022b; Study 2)	1	1	1	1	1	1	N/A	1	1	1	1	1	1	N/A	1	1	1	1	1	1	19
Smoker and March (2017)	1	1	0	1	1	1	0	1	0	1	1	1	0	0	1	1	1	1	DK	DK	13
Tetreault et al. (2021)	1	1	0	1	0	0	N/A	1	1	1	1	1	N/A	N/A	1	1	1	1	1	1	17

*Note.* Items scored “no”  = 0; Items scored “yes” = 1 except where * indicates reverse-scored items. Items marked N/A were awarded one point for overall rating. Item: 1. Were the aims/objectives of the study clear?; 2. Was the study design appropriate for the stated aim(s)? 3. Was the sample size justified?; 4. Was the target/reference population clearly defined?; 5. Was the sample frame taken from an appropriate population base so that it closely represented the target/reference population under investigation?; 6. Was the selection process likely to select subjects/participants that were representative of the target/reference population under investigation?; 7. Were measures undertaken to address and categorize nonresponders?; 8. Were the risk factors and outcome variables measured appropriate to the aims of the study?; 9. Were the risk factor and outcome variables measured correctly using instruments/measurements that have been trialed, piloted, or published previously?; 10. Is it clear what was used to determine statistical significance and/or precision estimates? (e.g., *p*-values and confidence intervals); 11. Were the methods (including statistical methods) sufficiently described to enable them to be repeated?; 12. Were the basic data adequately described?; 13. Does the response rate raise concerns about nonresponse bias?; 14. If appropriate, was information about nonresponders described?; 15. Were the results internally consistent?; 16. Were the results presented for all the analyses described in the methods?; 17. Were the authors’ discussions and conclusions justified by the results?; 18. Were the limitations of the study discussed?; 19. Were there any funding sources of conflicts of interest that may affect the authors’ interpretation of the results?; 20. Was ethical approval or consent of participants attained? N/A = not applicable; DK = don’t know.

## Meta-Analysis

### Overall Relationship

**Figure 2.**
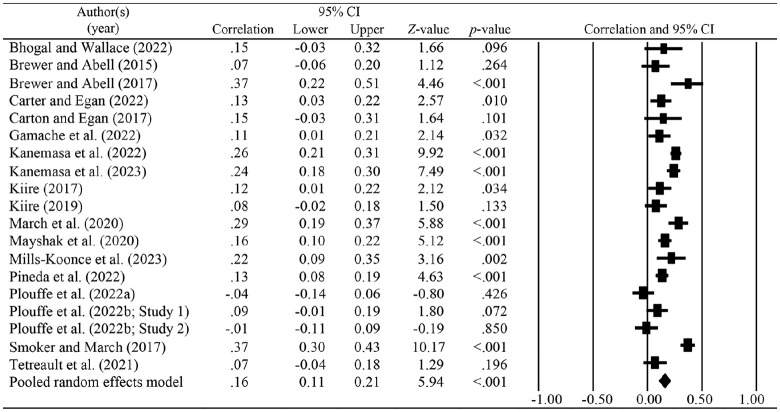
Random-effects meta-analysis and forest plot for overall relationship.

Figure 2 displays the forest plot for the overall meta-analysis. A total of 19 effect sizes were included in the overall random-effects meta-analysis revealing a significant, small, positive relationship between Machiavellianism and IPV perpetration (*r* = .16, 95% CI [0.11, 0.21], *p* < .001). Heterogeneity between the studies was high (*Q* = 108.64, *p* < .001, *I*^2^ = 83%, *T*^2^ = .01).

### Machiavellian Subtypes (Views and Tactics)

All included studies operationalized Machiavellianism as a unidimensional construct and did not investigate the Machiavellianism subtypes: views and tactics. Thus, it was not possible to carry out the second aim of this review which was to determine whether the strength of the relationship between Machiavellianism and IPV perpetration differed between these two subtypes.

### IPV Type

Table 3 contains a summary of the IPV-type subgroup analysis which revealed a significant, weak, positive relationship between Machiavellianism and cyber IPV perpetration (*r* = .25, 95% CI [0.17, 0.32], *p* < .001), psychological IPV perpetration (*r* = .20, 95% CI [0.15, 0.24], *p* < .001), and sexual IPV perpetration (*r* = .10, 95% CI [0.02, 0.19], *p* = .020). No significant relationship was found between physical IPV perpetration and Machiavellianism (*r* = .05, 95% CI [−0.01, 0.11], *p* = .072). Overall, the strength of the relationship between Machiavellianism and IPV perpetration differed between IPV types (*p* = .002). Cyber, psychological, and sexual IPV had significantly larger effect sizes than physical IPV. There was high heterogeneity for cyber IPV, and low to medium heterogeneity for all other IPV types.

**Table 3.** Subgroup Analyses Presenting Effect Size Estimates for Each IPV Type and Gender.

Subgroup	*k*	Effect size (*r*)	95% CI		*Heterogeneity*			
			Lower, Upper	*p*	*Q*	*p*	*I* ^2^	*T* ^2^
IPV type								
Cyber IPV	4	.25	[.17, .32]	<.001	30.64	<.001	90.21	.01
Psychological IPV	12	.20	[.15, .24]	<.001	28.82	.002	61.83	.01
Sexual IPV	4	.10	[.02, .19]	.020	.96	.812	0.00	.01
Physical IPV	9	.05	[−.01, .11]	.072	11.33	.184	29.38	.01
Gender								
Women	6	.19	[.09, .29]	<.001	19.85	<.001	74.80	.01
Men	4	.10	[−.03, .23]	.134	10.64	.014	71.82	.01

*Note.* df = number of studies less 1. IPV = intimate partner violence.

### Gender

Table 3 contains a summary of the gender subgroup analysis which revealed a significant, weak, positive relationship between Machiavellianism and IPV perpetration for women (*r* = .19, 95% CI [0.09, 0.29], *p* < .001), though no significant relationship was found

for men (*r* = .10, 95% CI [−0.03, 0.23], *p* = .134). However, there was no significant difference in the strength of the relationship between Machiavellianism and IPV perpetration for men and women (*p* = .301). Heterogeneity was medium for both women and men.

## Publication Bias

**Figure 3.**
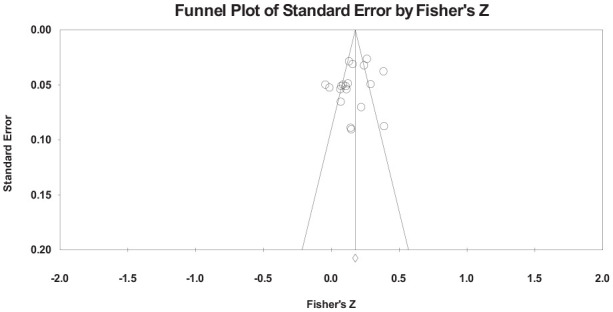
Funnel plot for all included effect sizes.

Figure 3 shows the funnel plot for the main analysis which was inspected visually to assess for publication bias. There was some mild asymmetry however no study unduly impacted the overall results as indicated by a leave-one-out analysis. When each study was removed, a significant, weak, positive relationship remained between Machiavellianism and IPV perpetration with effect sizes ranging from *r* = .14 to *r* = .17. Further, Egger’s regression test was nonsignificant, indicating no evidence of bias (Egger’s intercept = −2.00, *p* = .239). A Duval and Tweedie (2000) trim-and-fill analysis showed that no studies were required to be trimmed from the left of the funnel plot, and three to the right. When these three studies were removed, the adjusted effect size remained significant, weak, and positive (*r* = .19, 95% CI [0.18, 0.21]), suggesting that no bias was detected. Finally, the classic failsafe *N* method indicated that 1,104 nonsignificant studies would need to be missing to affect the findings of this meta-analysis. Given only 19 studies were identified for inclusion, it was considered extremely unlikely that 1,104 studies were either unpublished or otherwise undetected by the systematic searching of this review.

## Discussion

This review aimed to estimate the strength of the relationship between Machiavellianism and IPV perpetration. Nineteen studies were included in the random-effects meta-analysis which, as expected, revealed a significant, small, positive relationship between Machiavellianism and IPV perpetration. That is, as self-reported Machiavellianism traits increased, so did IPV perpetration. This finding builds on the current literature examining the relationships between IPV perpetration and the Dark Triad traits by confirming that alongside narcissism (Oliver et al., 2023) and psychopathy (Robertson et al., 2020), Machiavellianism is also linked to violent behavior perpetrated in intimate partnerships. Although the association was small, this finding aligns with previous studies showing a relationship between Machiavellianism and violence perpetrated against intimate partners (Kanemasa et al., 2022), as well as in other dynamics such as violence perpetrated against peers (Sehar & Fatima, 2016), and strangers (Pailing et al., 2014).

The second aim was to determine whether the strength of the relationship differed across the Machiavellianism subtypes of views and tactics. It was expected that IPV perpetration would be more strongly correlated with Machiavellian tactics compared to Machiavellian views. However, because all studies operationalized Machiavellianism as a unidimensional construct and did not examine these subtypes, this aim could not be addressed.

The third aim was to examine whether there were differences in the strength of the relationship across types of IPV perpetrated. As hypothesized, it was found that the strength of the relationship between IPV perpetration and Machiavellianism was dependent on the IPV type examined. A statistically significant, small, positive relationship was found between Machiavellianism and *cyber, psychological*, and *sexual* IPV respectively. However, there was no significant relationship between Machiavellianism and *physical* IPV. Indeed, the strength of the relationship between Machiavellianism and cyber, psychological, and sexual IPV was significantly greater than for physical IPV.

Taken together, these findings indicate that Machiavellianism has a stronger association with nonphysical types of IPV. Although sexual IPV has a physical component, it is important to highlight that some of the tools used to measure this behavior incorporated sexually coercive tactics of a psychological nature. For example, Brewer and Abell (2015) used their Sexual Coercion in Intimate Relationships Scale which examines threats and other verbally manipulative behaviors, whereas the physical IPV measures focused solely on physical violence. One potential explanation for the differences among IPV types may be owing to the inherent characteristics of Machiavellianism. Machiavellianism is marked by calculated behavior to achieve one’s goals in a way that avoids reputation damage (Jones & Paulhus, 2010; Kiire, 2017). Thus, overt physical violence may be a less effective tool for people high in Machiavellianism compared to the covert manipulation tactics involved in more emotionally manipulative based IPV types such as cyber, psychological, and sexual IPV. This notion is supported by Monaghan et al. (2016) who suggest that the typical externalizing behavior associated with Machiavellianism involves goal-focused social manipulation rather than direct, aggressive violence.

The fourth and final aim was to investigate whether the relationship between Machiavellianism and IPV perpetration differed across the gender of the IPV perpetrator. A significant, small, positive relationship was observed between Machiavellianism and IPV perpetration for women, but not men. However, there was no statistically significant difference in the size of the relationship between men and women. This may be due to the small number of effect sizes in this analysis which included more women samples (*k* = 6 women, *k* = 4 men). Nevertheless, these findings align with previous research showing parity among genders in relation to IPV perpetration risk factors (Moffitt et al., 2001), and reinforces the arguments of Tetreault et al. (2021) who advocate for a gender-inclusive approach to IPV research. A summary of the critical findings is presented in Table 4.

**Table 4.** Critical Findings.

Machiavellianism and IPV perpetration
• There is a significant, small, positive relationship between Machiavellianism and IPV perpetration.
Machiavellianism and IPV type
• The relationship between Machiavellianism and IPV perpetration was moderated by IPV type.
• There is a significant, small, positive relationship between Machiavellianism and cyber, sexual, and psychological IPV. • There is no significant relationship between Machiavellianism and physical IPV.
• The strength of the relationship between Machiavellianism and cyber, psychological, and sexual IPV was significantly greater than for physical IPV.
• Machiavellianism has a stronger association with nonphysical types of IPV.
Machiavellianism and IPV perpetration by men and women
• There is a significant, small, positive relationship between Machiavellianism and IPV perpetration for women, but not men. However, there is no significant difference in the strength of the relationship between Machiavellianism and IPV perpetration between men and women.

*Note*. IPV = intimate partner violence.

### Limitations of Included Studies and Present Review

This review and the included studies present several limitations. First, self-report measures were used as the primary data source among most of the included studies for both Machiavellianism and IPV perpetration. These are two socially undesirable constructs, and under-reporting may have therefore impacted the findings of the included studies and the conclusions drawn in this review. This is particularly true of Machiavellianism which, by its very nature, is likely to lead individuals to misrepresent themselves. Likewise, all included studies recruited volunteer participants from community and undergraduate samples, thus it is possible that participants with high levels of Machiavellianism, or those who engage in more extreme types of IPV perpetration, may not have been captured in this review. Although the study criteria of the present meta-analysis allowed for such participants, as well as for objective measures of IPV such as court reports or police records, no such studies were identified. These factors could be partially mitigated in future studies by targeting convicted offenders, employing more objective IPV measures, or by recruiting couples where IPV data can be collected from both parties, rather than relying solely on perpetrator reports (Kan & Feinberg, 2010).

There was also variability across the studies in the methods used to assess IPV perpetration which likely accounts for the heterogeneity in the present review. This inconsistency in defining, measuring, and examining IPV is a longstanding criticism of the research where inconsistencies have resulted in a lack of clarity about the causes and outcomes of IPV (Bagwell-Gray et al., 2015). As Bagwell-Gray et al. (2015) highlight and as this review reinforces, there is a need to generate a consistent framework for defining and measuring IPV to ensure that researchers, practitioners, and the general population are aligned in their understanding. Further to this, there was a limited number of studies examining cyber and sexual IPV types. This, again, is potentially due to differences in how these IPV types are defined and measured, and for cyber IPV, due to the recency of the measures used to assess this form of IPV. Moreover, few studies reported correlations separately by gender. Future studies should examine these factors to clarify their effect on the relationship between IPV perpetration and Machiavellianism.

As earlier highlighted, Machiavellianism was operationalized as a unidimensional construct among all included studies. This is perhaps unsurprising given the recency of the bifactor model of Machiavellianism. Nonetheless, this was a shortfall of the studies included in the review given it has been argued that the trait comprised two theoretically distinct subtypes (views and tactics) which correlate differently with a range of external variables (Monaghan et al., 2016). As a result, this review was unable to provide insight into how each of the Machiavellian dimensions uniquely relates to IPV perpetration.

Additionally, in terms of sample demographics, all studies included in this review recruited predominantly heterosexual samples and a large portion did not report the ethnicity or education level of participants. Of those that did, most were white, had accessed tertiary education, and were from more economically developed countries. As such, there is a lack of diversity in the sexuality, ethnicity, and education level of the participants contained in this meta-analysis and it is therefore not an accurate representation of the broader population. It remains unclear whether the observed relationships between Machiavellianism and IPV perpetration are extant among diverse populations and more research is needed to clarify this.

### Implications and Future Research

These findings suggest that Machiavellianism may be relevant to clinical practice when providing psychological support to couples or individuals where IPV has been perpetrated. Although most individuals high in dark personality traits may be resistant to change, research such as that of Hudson (2023) has shown that the Machiavellianism trait and its expression can be indirectly reduced through targeting other traits, namely agreeableness. As such, interventions focused on agreeableness may be one mechanism for reducing the expression of Machiavellianism, and thus IPV perpetration when working clinically with perpetrators. However, as Hudson (2023) emphasizes, for changes in personality traits and the associated behaviors to occur, a level of intent from the individual to actually change is required. Given some people high in Dark Triad traits are able to experience empathy (Heym et al., 2020), it is possible that the provision of screening tools and psychoeducation about the Dark Triad traits may serve to build insight and motivation to reduce the expression of Machiavellianism and thus IPV perpetration. However, there is a clear need for further research that examines the causal pathways between Machiavellianism and IPV perpetration to determine whether such approaches may be effective.

Psychoeducation about the expression of Dark Triad traits may also assist those who have been subjected to or are potential victims of IPV. For example, awareness about the relationship between Machiavellianism and IPV perpetration may empower individuals in a violent relationship to identify concerning behavior which could help to improve their safety within the dynamic. Likewise, such knowledge may enable those seeking an intimate partner to be informed when making decisions about the individuals they select to date. Further, it is not uncommon for those who have been exposed to IPV to blame themselves (Camp, 2022). The findings of this meta-analysis may therefore provide a framework for individuals to better understand IPV perpetration, which in turn, may help to reduce feelings of shame and validate experiences.

Finally, as highlighted throughout, Monaghan et al. (2016) argue that a bifactor model of Machiavellianism provides a more sophisticated understanding of the nature of Machiavellianism. As such, the bifactor model should be employed in future studies to improve the broader understanding of Machiavellianism, and its association with antisocial behaviors such as IPV perpetration. A summary of the implications for practice, policy, and research is presented in Table 5.

**Table 5.** Implications of the Present Meta-analysis for Practice, Policy, and Research.

Implications for practice
• When working clinically with perpetrators of IPV, the provision of screening tools and psychoeducation may be one mechanism to build insight and motivation to reduce expression of Machiavellianism characteristics.
• Providing psychoeducation about the relationship between Machiavellianism and IPV perpetration may serve to build awareness about concerning characteristics or behaviors that may place an individual at risk of harm. This knowledge may assist individuals already in a violent relationship to help improve safety within the dynamic, while also enabling individuals to be better informed about concerning traits when selecting an intimate partner.
Implications for policy
• Policymakers should consider the variety of ways in which IPV is perpetrated and establish legislation that appropriately captures the perpetration of such violence, including psychological and cyber IPV in particular.
Implications for research
• Researchers should use tools that measure IPV perpetration in more objective ways such as through court records, police reports, or by gathering data from both parties within a couple, rather than relying on self-reporting by perpetrators.
• When assessing Machiavellianism in any context, researchers should use a bifactor model of Machiavellianism given the unique nature of the Machiavellianism views and tactics subtypes.
• Future researchers should aim for a consistent approach to defining, measuring, and examining IPV which, at a minimum, captures the different subtypes, including physical, psychological, sexual, and cyber IPV.

*Note*. IPV = intimate partner violence.

## Conclusions

In sum, this review revealed that Machiavellianism has a significant, small, positive relationship with IPV perpetration. This relationship appears to be influenced by the type of IPV being examined. Machiavellianism was significantly correlated with *cyber, psychological*, and *sexual* IPV, though no significant association was found for *physical* IPV. Although the association between Machiavellianism and IPV perpetration was significant for women but not men, no statistically significant difference in this relationship was found between the two genders. Collectively, these findings confirm that like narcissism and psychopathy, Machiavellianism is linked to IPV perpetration. Accordingly, the provision of screening tools and psychoeducation about Machiavellianism may be of value to improve the insight and treatment outcomes of perpetrators. These findings may also validate the experiences of those who have been subjected to IPV by providing a framework to better understand perpetrator behavior. Given there is a current deficit in research targeting reductions in the Dark Triad traits and the associated antisocial behavior, further studies are needed to confirm effective interventions for both perpetrators of IPV, and those exposed to it.

## Supplemental Material

sj-docx-1-tva-10.1177_15248380241270027 – Supplemental material for Machiavellianism and Intimate Partner Violence Perpetration: A Systematic Review and Meta-Analysis Supplemental material, sj-docx-1-tva-10.1177_15248380241270027 for Machiavellianism and Intimate Partner Violence Perpetration: A Systematic Review and Meta-Analysis by Lisa K. White, Natasha Valos, Xochitl de la Piedad Garcia and Megan L. Willis in Trauma, Violence, & Abuse

## Notes

1. All included studies aside from four (Carter & Egan, 2022; Carton & Egan, 2017; Kiire, 2017; Mayshak et al., 2020) reported on gender (men/women) rather than biological sex (female/male).
