# Supplementary material for: Machiavellianism and Intimate Partner Violence Perpetration: A Systematic Review and Meta-Analysis
Source: Trauma Violence Abuse. 2024 Aug 20;25(5):4159–72. doi: 10.1177/15248380241270027 (PMC11555792; doi:10.1177/15248380241270027)
Supplement: sj-docx-1-tva-10.1177_15248380241270027 – Supplemental material for Machiavellianism and Intimate Partner Violence Perpetration: A Systematic Review and Meta-Analysis [file sj-docx-1-tva-10.1177_15248380241270027.docx]

**Supplementary Information**

**Search Strategy**

| Database | Search concept one: Intimate partner violence | Search concept two: Machiavellianism |
| --- | --- | --- |
| Medline Complete and PsycInfo | TI (“intimate partner*” OR “interpersonal violence” OR “relationship quality” OR “spous* violence” OR “spous* abuse” OR “spous* assault” OR “batter*” OR “marital violence” OR “marital abuse” OR “marital assault” OR “domestic violence” OR “domestic abuse” OR “domestic assault” OR “family violence” OR “family abuse” OR “family assault” OR “dating violence” OR “dating abuse” OR “dating assault” OR “psychological violence” OR “psychological abuse” OR “emotional violence” OR “emotional abuse” OR “coerci*” OR “gaslight*” OR “sexual violence” OR “sexual abuse” OR “sexual assault” OR “physical violence” OR “physical assault” OR “physical abuse” OR “cyber*”) OR AB (“intimate partner*” OR “interpersonal violence” OR “relationship quality” OR “spous* violence” OR “spous* abuse” OR “spous* assault” OR “batter*” OR “marital violence” OR “marital abuse” OR “marital assault” OR “domestic violence” OR “domestic abuse” OR “domestic assault” OR “family violence” OR “family abuse” OR “family assault” OR “dating violence” OR “dating abuse” OR “dating assault” OR “psychological violence” OR “psychological abuse” OR “emotional violence” OR “emotional abuse” OR “coerci*” OR “gaslight*” OR “sexual violence” OR “sexual abuse” OR “sexual assault” OR “physical violence” OR “physical assault” OR “physical abuse” OR “cyber*”) | TI (“Machiavellian*” OR “dark triad” OR “dark tetrad” OR “dark personalit*”) OR AB (“Machiavellian*” OR “dark triad” OR “dark tetrad” OR “dark personalit*”) |
| Scopus | (TITLE(“intimate partner*” OR “interpersonal violence” OR “relationship quality” OR “spous* violence” OR “spous* abuse” OR “spous* assault” OR “batter*” OR “marital violence” OR “marital abuse” OR “marital assault” OR “domestic violence” OR “domestic abuse” OR “domestic assault” OR “family violence” OR “family abuse” OR “family assault” OR “dating violence” OR “dating abuse” OR “dating assault” OR “psychological violence” OR “psychological abuse” OR “emotional violence” OR “emotional abuse” OR “coerci*” OR “gaslight*” OR “sexual violence” OR “sexual abuse” OR “sexual assault” OR “physical violence” OR “physical assault” OR “physical abuse” OR “cyber*”) OR ABS (“intimate partner*” OR “interpersonal violence” OR “relationship quality” OR “spous* violence” OR “spous* abuse” OR “spous* assault” OR “batter*” OR “marital violence” OR “marital abuse” OR “marital assault” OR “domestic violence” OR “domestic abuse” OR “domestic assault” OR “family violence” OR “family abuse” OR “family assault” OR “dating violence” OR “dating abuse” OR “dating assault” OR “psychological violence” OR “psychological abuse” OR “emotional violence” OR “emotional abuse” OR “coerci*” OR “gaslight*” OR “sexual violence” OR “sexual abuse” OR “sexual assault” OR “physical violence” OR “physical assault” OR “physical abuse” OR “cyber*”)) | (TITLE(“Machiavellian*” OR “dark triad” OR “dark tetrad” OR “dark personalit*”) OR ABS (“Machiavellian*” OR “dark triad” OR “dark tetrad” OR “dark personalit*”)) |
| Web of Science | “intimate partner*” OR “interpersonal violence” OR “relationship quality” OR “spous* violence” OR “spous* abuse” OR “spous* assault” OR “batter*” OR “marital violence” OR “marital abuse” OR “marital assault” OR “domestic violence” OR “domestic abuse” OR “domestic assault” OR “family violence” OR “family abuse” OR “family assault” OR “dating violence” OR “dating abuse” OR “dating assault” OR “psychological violence” OR “psychological abuse” OR “emotional violence” OR “emotional abuse” OR “coerci*” OR “gaslight*” OR “sexual violence” OR “sexual abuse” OR “sexual assault” OR “physical violence” OR “physical assault” OR “physical abuse” OR “cyber*” (Title) or “intimate partner*” OR “interpersonal violence” OR “relationship quality” OR “spous* violence” OR “spous* abuse” OR “spous* assault” OR “batter*” OR “marital violence” OR “marital abuse” OR “marital assault” OR “domestic violence” OR “domestic abuse” OR “domestic assault” OR “family violence” OR “family abuse” OR “family assault” OR “dating violence” OR “dating abuse” OR “dating assault” OR “psychological violence” OR “psychological abuse” OR “emotional violence” OR “emotional abuse” OR “coerci*” OR “gaslight*” OR “sexual violence” OR “sexual abuse” OR “sexual assault” OR “physical violence” OR “physical assault” OR “physical abuse” OR “cyber*” (Abstract) | “Machiavellian*” OR “dark triad” OR “dark tetrad” OR “dark personalit*” (Title) or “Machiavellian*” OR “dark triad” OR “dark tetrad” OR “dark personalit*” (Abstract) |

*Note.* Searches were conducted with “AND” used as a conjoining Boolean operator between the two search concepts
